# Randomized, Double-Blind, and Placebo-Controlled Trial of Clenbuterol in Denervated Muscle Atrophy

**DOI:** 10.5402/2011/981254

**Published:** 2011-08-15

**Authors:** Guang-Liang Jiang, Yu-Dong Gu, Li-Yin Zhang, Li-Ying Shen, Cong Yu, Jian-Guang Xu

**Affiliations:** ^1^Department of Hand Surgery, Huashan Hospital of Fudan University, Shanghai 200040, China; ^2^Clinical Development, Neurology & Pain, R&D, Allergan Inc., 2525 Dupont Dr., Irvine, CA 92612, USA

## Abstract

*Objectives*. *β*_2_-adrenergic agonists, such as clenbuterol, have been shown to promote the hypertrophy of healthy skeletal muscles and to ameliorate muscle wasting in a few pathological conditions in both animals and humans. We intended to investigate the clinical efficacy of clenbuterol on attenuating denervation-induced muscle atrophy. *Methods*. A double-blind, placebo-controlled, parallel, and randomized trial was employed. 71 patients, suffering from brachial plexus injuries, were given either clenbuterol (60 *μ*g, bid) or placebo for 3 months. Before and at the end of the study, patients were given physical examinations, biopsies of biceps brachii, electromyograms (EMGs), and other laboratory tests. *Results*. Compared with placebo treatment, clenbuterol significantly mitigated the decreases in cross-sectional areas of type I and II muscle fibers and alleviated the reduction in fibrillation potential amplitudes, without any adverse effects. *Conclusions*. Clenbuterol safely ameliorated denervated muscle atrophy in this cohort; thus larger clinical studies are encouraged for this or other *β*_2_ agonists on denervation-induced muscle atrophy.

## 1. Introduction

Selective beta-2 adrenergic agonists have been shown to increase skeletal muscle mass and function under physiological conditions in a variety of species. This is attributable to their roles in controlling protein synthesis and degradation, via G*α*_s_ and G*βγ* coupled signalings and Ca^2+^/ubiquitin-dependent pathways, respectively [1]. *β*2 agonists also ameliorate animal muscle wasting in denervation, amyotrophic lateral sclerosis, muscular dystrophy, disuse, aging, and myocardial unloading models [2–8].

In patients with immobilization conditions or muscular dystrophy, *β*2 agonists, such as clenbuterol, increase lean body mass and enhance skeletal muscle functions [9–11]. In addition, clenbuterol (up to 720 *μ*g/day) promotes myocardial recovery in patients with myocardial unloading atrophy resulting from applications of left ventricular assist devices [12–14].

In comparison with other *β*2 agonists, clenbuterol, a long-acting and functionally selective agent, is the most effective drug in preventing muscle wasting, with the least adverse effects [13, 15–18]. However, the clinical efficacy of clenbuterol on denervated muscle atrophy has not yet been reported; denervation-induced muscle atrophy remains an unmet medical need. Also muscle atrophy status ultimately determines the functional recovery of repaired peripheral nerve injuries [19]. Based on *β*2 agonists' roles in promoting hypertrophy of intact skeletal and cardiac muscles, and in preventing muscle wasting in preclinical studies, we hypothesized that clenbuterol may mitigate denervated muscle atrophy in humans. The current pilot trial tested clenbuterol's effects on retarding denervation-induced muscle atrophy in patients.

## 2. Materials and Methods

### 2.1. Patients

71 patients were recruited to this study at the Hand Surgery Department of Huashan Hospital, Fudan University in Shanghai. Entering criteria: (1) patients had loss of innervation in the biceps brachii for more than one month, and diagnosis was achieved through the combination of history, physical examinations, electromyogram (EMG), and examinations during surgeries. (2) The loss of innervation must have resulted solely from traumatic injuries to the brachial plexus, namely, C5/6, C5/6/7, or C5-T1 injuries, and not from other causes, such as virus infections or autoimmune diseases. (3) Besides the trial, all studied patients had phrenic nerves transferred to musculocutaneous nerves, in addition to various other nerve transfers. (4) All patients were routinely given oral Vitamin B6 (10 mg tid) and Dibazolum (10 mg tid) to facilitate nerve regeneration during the trial period. (5) The participating patients had no central nervous system injuries or other diseases and were not in any other trials. (6) All patients signed the informed consents. Excluding criteria: (1) ischemia or traumatic compressions occurring directly on the biceps brachii or musculocutaneous nerves; (2) pregnant or breast feeding women; (3) patients on *β*-blockers for other diseases; (4) patients who had experienced serious adverse events from other *β*-agonists before; (5) patients, who were not strictly compliant in taking trial drugs and completing the adjunctive therapies as above, were not analyzed; (6) patients, who did not revisit at their scheduled time, were not analyzed.

### 2.2. Design

Patients were recruited from 5 therapeutic teams led by independent investigators at the department. They were labeled with case numbers and randomly assigned into clenbuterol or placebo groups. The codes were stored separately. It was only referred to by the organizer at the end of the study or when a severe adverse event occurred. Neither the therapeutic team members nor patients knew which group a patient was assigned to. The EMG examination staffs and the processor/evaluator of the biopsy samples were masked from treatment allocations. Clenbuterol was formulated as 60 *μ*g/10 mL in sealed glass tubes by pharmacists. A total of 20,000 tubes of clenbuterol or placebo were made, respectively. Shelf stability at 4°C was tested to guarantee the constancy of the active pharmacological ingredient over the entire study period. Each patient was prescribed 200 tubes of clenbuterol or placebo and was asked to take the medicine orally 60 *μ*g twice per day for 3 months (~2 *μ*g/kg/day), without food interference. Significant muscle atrophy was observed during 3-month period after denervation [20]. And also meaningful nerve regeneration may have not occurred in the majority of cases during the 3-month period, thus it will not confound the study medicine efficacy. A risk management plan was also formed to safeguard the patients. During hospitalization, patients in both groups were monitored daily for any discomfort in the first week of drug administration. It was planned to stop the treatments if patients demonstrated either allergic responses or intolerable muscle tremors, severe diarrhea, cardiac arrhythmia, or other unexpected adverse reactions. They were all encouraged to call in to report any adverse events during the 3-month trial. In addition, all patients accepted the conventional surgical nerve repairs as mentioned above. Nevertheless, the patients were not given any other medicines known to affect muscle atrophy. Patients in both groups were required for assessments at 3 months after the trial's initiation. During the follow-up visit, the leftover drugs were counted to verify compliance, and a retrospective survey was conducted to find out any potential adverse effects. The study protocol was approved by the Institutional Review Board at Huashan Hospital. The planned primary outcome measure was changes in muscle cross-sectional fiber sizes over the treatment period of 3 months, which is a valid and basic method to assess muscle atrophy. The secondary outcome measures were changes in fibrillation potential amplitudes and the percentage of biceps reinnervation after 3 months treatment.

### 2.3. General Laboratory Evaluations

Before the trial, each patient was admitted and given a set of laboratory evaluations including a 12-lead electrocardiogram, chest X-ray, pulmonary function test, and several liver function studies. Blood urea nitrogen, creatinine, bleeding time, prothrombin time, glucose, calcium, phosphorus, electrolytes, and complete blood counts with differentials were also recorded. By the end of the trial, all patients repeated the above examinations regardless of their groups.

### 2.4. EMG Examination

At the commencement and conclusion of the trial, each patient was given EMG examinations with a Dantec Neuromatic-2000 M (Denmark) electromyography in an air-conditioned room, after adapting to a constant temperature for 10 minutes. Patients were given a full set of conventional EMG examinations on each nerve of both upper extremities, including sensory and motor nerve conduction speeds, maximum and evoked motor unit action potentials (MUAPs), and somatosensory evoked potentials (SEPs) at different segments of the brachial plexus and terminal branches. The results were compared between both sides by EMG professionals. If there were any doubts about the EMG diagnosis, an intraoperational EMG examination was conducted after exposing the root, trunk, cord, or some proximal branches of the brachial plexus. If no MUAP or SEP was detected, fibrillation potential amplitudes were then recorded, via a concentric needle electrode inserted perpendicularly into at least three spots around the midpoints of the biceps brachii. Only the one with the largest amplitude and biphasic or triphasic shape was analyzed when several fibrillation firings were recorded, because the largest amplitudes best reflect the sizes of muscle fibers closest to the needle tip [20, 21]. The complete EMG data for the brachial plexus injury diagnosis will not be listed in the present paper, as those are not as highly relevant as fibrillation potential amplitudes in the intended trial.

### 2.5. Muscle Biopsy

All 71 patients were given muscle biopsies at the midpoints of the biceps brachii during nerve repair operations right before the start of the trial. Biopsy samples were covered with talc powder and snap frozen in isopentane precooled in liquid nitrogen. 10 *μ*m thick fresh cryostat sections were made on a cryostat microtome at −20°C and mounted on slides. Myofibrillar adenosine triphosphatase staining at either pH 4.6 or 9.4 was performed on each sample to show fiber types [22]. Fiber cross-sectional areas were measured on approximately 300 fibers from three different view fields with a computed image analysis system; the average was calculated for every fiber type in each sample. By the end of the trial, if EMG and physical examinations showed no signs of reinnervation of the biceps brachii, patients from both groups were asked to have repetitive biopsies of the biceps brachii if the patients were fully compliant to study instructions. The muscle samples were processed as before.

### 2.6. Statistical Analysis

All patient information and examinations were put into SPSS software. The changes in fibrillation potential amplitudes and cross-sectional areas over the trial period in each patient were used for statistical analysis. *P* < 0.05 was taken as statistical significance.

## 3. Results

### 3.1. Characteristics of the Study Subjects

**Figure 1:**
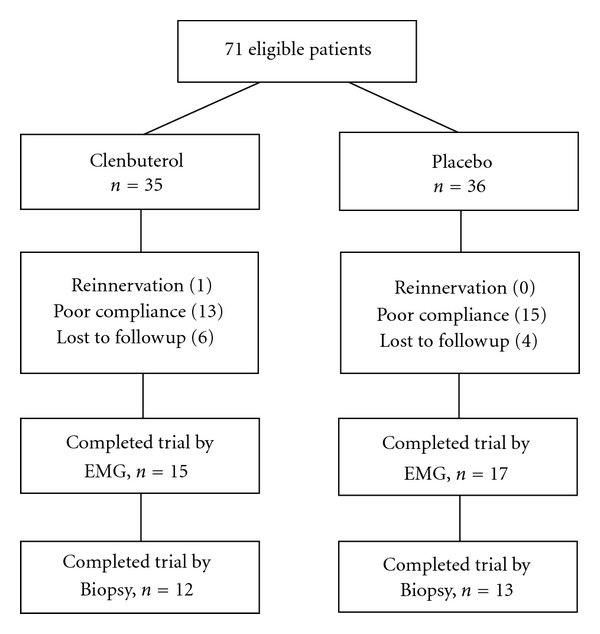
Study design and follow-up flow chart.

35 patients were randomized into clenbuterol group, and 36 patients were allocated into placebo group. Poor compliance (not taking medicine as prescribed and delayed followup visit) and lost to follow-up accounted 19 cases in clenbuteral or placebo group, respectively. One patient in the clenbuterol group showed signs of reinnervation of the biceps brachii (M2) 3 months after the phrenic nerve transfer. This was determined through physical and EMG examinations. 15 patients in clenbuteral group and 17 patients in the placebo group completely obeyed the guidelines of the 3-month trial with no indications of reinnervation of their biceps brachii (Figure 1). Among them, 12 patients in the clenbuterol group and 13 patients in the placebo group accepted repetitive muscle biopsies. Patients were mainly young people suffering from motorcycle accidents or other traumas. There were no significant differences in ages between the two treatment groups. Denervation time before trial enrollment was around 5 months, a period not significantly different between the groups (Table 1). All patients accepted similar surgical procedures and conventional adjunctive therapies. These key characteristics made the patients in both groups comparable.

### 3.2. Efficacy on Retarding Muscle Atrophy

**Figure 2:**
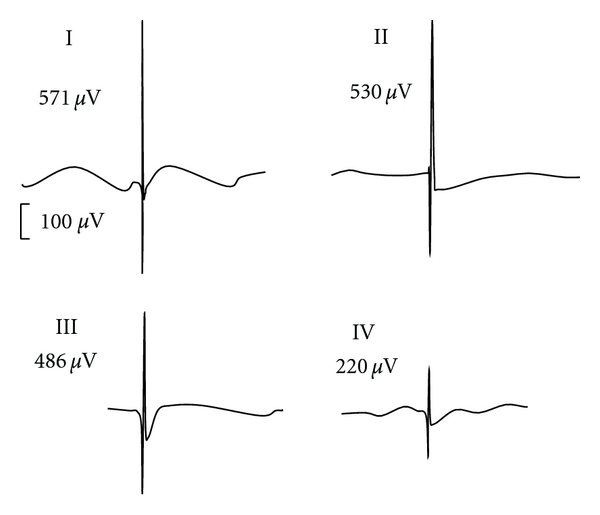
Representative EMGs prior to (I) and after (III) clenbuterol treatment, from a 27-year-old male with right brachial plexus injury for two months at the time of enrollment; (II) and (IV) are representative EMGs prior to (II) and after (III) placebo treatment of a 21-year-old male with left brachial plexus injury for one and a half month before the trial.

Denervated muscles produce spontaneous, repetitive single-fiber discharges that are presented as fibrillation potentials and are detectable by electromyography. The amplitudes of fibrillation potentials correlate well with muscle fiber sizes [20, 21]. Patients treated with clenbuterol had an average reduction of 57 *μ*V in the amplitudes of fibrillation potentials, while patients accepting the placebo had a 4-time greater decrease over the same period of time (Table 2, Figure 2). 

**Figure 3:**
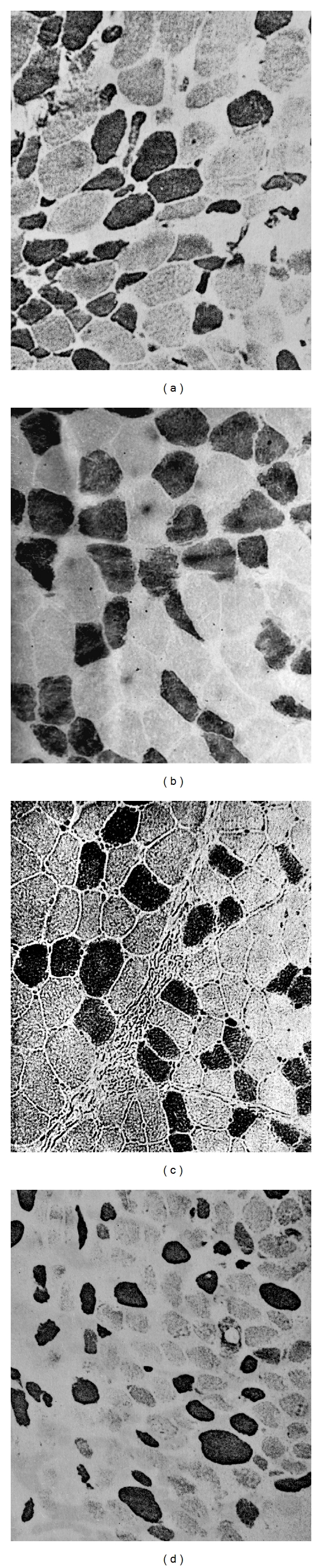
Representative images of ATPase staining at pH 9.4 to show darkly stained type II fibers on biopsied biceps brachii (100x magnification). (a) is from a 26-year-old male with right brachial plexus injury for 3 months, and (c) is from the same patient after 3-month clenbuterol treatment. (b) is from another 26-year-old male with right brachial plexus injury for 3 months, and (d) is from the same patient after 3-month placebo treatment.

In the present study, denervated muscle fibers atrophied over time, and type I fibers had less decrease in cross-sectional areas than type II, regardless of treatments, similar to results reported elsewhere [20]. The reductions of cross-sectional areas in type I and II fibers were 413 and 512 *μ*m^2^, in clenbuterol-treated patients. Yet, those decreases in fiber sizes were correspondingly 66% and 60% greater in placebo-treated patients (Table 2, Figure 3).

### 3.3. Adverse Event Monitoring

Out of all the patients, just one patient felt transient muscle tension 2 days after the clenbuterol administration. No special intervention was given except observation and followups; the discomfort disappeared after attention distraction for half a day. The electrocardiogram (EKG) of one patient recorded newly occurring sinus arrhythmia and bradycardia after 3 months of clenbuterol administration. He had no discomfort complaints. 

Six patients in the clenbuterol group had EKG abnormalities before enrollment, namely, sinus arrhythmia (2 cases), sinus bradycardia (1 case), incomplete right bundle branch block (1 case), right ventricular high voltage (1 case), and counterclockwise rotation of the heart (1 case). They all had no corresponding symptoms and required no treatments per consulting with cardiologists. Clenbuterol treatment for 3 months did not change their EKG presences. 

Seven patients in the placebo group recorded slight abnormalities in EKG (4 cases), glucose (2 cases), and creatinine (1 case) by the end of the study. There were no abnormalities in lung, liver functions, bleeding time, prothrombin time, electrolytes, or complete blood counts. No complaints of muscle tremors, tachycardia, dizziness, appetite changes, or diarrhea were reported over the trial period in either group.

## 4. Discussion

In the present study, we were able to recruit a special cohort of patients in their twenties. This helped to minimize the effects of aging on muscle atrophy and eliminate this potential confounding factor in the study, because spontaneous denervation occurs with aging [23, 24]. All patients had traumatic brachial plexus injuries with similar postinjury periods. Muscle fiber sizes decrease lineally over time during the first year [20]. In this trial, patients were observed mainly from the third to eighth month, the period of rapid atrophy. This time window might have facilitated disclosing clenbuterol's efficacy. Also, only one identical muscle of complete denervation was investigated in all of the patients, thus eliminating the differences in muscle fiber components concomitant with studying various or partially innervated muscles. Such similarities rendered the two groups to be comparable. The abundant patient population resulted mainly from motorcycle and construction accidents due to the transition of economic modernization in China. 

This is the first clinical study showing that clenbuterol at 120 *μ*g/day attenuated denervation-induced muscle atrophy in humans. It is reported that clenbuterol, at incremental doses from 120 to 720 *μ*g/day over 12 weeks, increases the mass and strength of the healthy skeletal muscles in man [13, 14]. Animal studies show that denervated skeletal muscles are 20 times more sensitive to clenbuterol than healthy muscles and the heart [25]. Therefore, 120 *μ*g/day of clenbuterol is believed to be a reasonably high dosage for the denervated muscles of patients. In the present study, the changes of fiber sizes and fibrillation potentials were used to evaluate the efficacy, aiding the avoidance of confounding factors resulting from variations in basal levels between individuals. Hence, it seems that fewer patients were required to reach a conclusion than the number needed by a study limited to just comparing endpoints.

In animal and large population studies, fibrillation potential amplitudes reflect fiber sizes of denervated muscles [20, 21, 26]. It was noted that clenbuterol treatment alleviated the reduction of fibrillation potential amplitudes by 300%, while atrophy of fiber sizes was only retarded by 66% or 60% in type I or II fibers, respectively. This discrepancy may be due to the fact that different muscle fibers were checked, particularly as muscle fiber sizes vary significantly even in one single biopsy sample of a denervated muscle (Figure 3). Only the largest fibrillation potential amplitudes were analyzed in this study, which probably originated from the large muscle fibers. Biopsies tended to collect a small bundle of muscle fibers, which reflects the overall status of muscle atrophy and should therefore be served as a gold standard. However, fibrillation potentials seem more sensitive in detecting the efficacy of therapeutics, which may consequently be used as an interim surrogate marker for future studies.

Reinnervation of muscle fibers, such as via axon regeneration following surgical repairs, often halts muscle atrophy and restores muscle functions [27]. Understanding such, we removed the patient with partial reinnervation of the biceps brachii from muscle atrophy analysis, to avoid interference with drug effects. Since the distance of nerve regeneration varies in patients with different limb lengths, we could not determine whether the case of reinnervation was due to clenbuterol treatment. Though, recent animal studies show that clenbuterol promotes peripheral nerve regeneration and prevents neuron loss in pathological conditions, via upregulating nerve growth factor, basic fibroblast growth factor and transforming growth factor-*β*1 [5, 28, 29]. We could not conclude if clenbuterol promotes nerve regeneration given the short observation period. To explode this, future studies could stratify patients by the demanding length of regeneration and by lengthening the treatment and observation time. 

Skeletal muscle contains all three *β*-adrenoceptor subtypes (*β*_1_, *β*_2_ and *β*_3_), with about a 10-fold greater proportion of the *β*_2_-adrenoceptor isoform than *β*_1_ or *β*_3_ receptors [30, 31]. The expression of subtype receptors remains to be checked under denervation condition in the future studies. Conversely, cardiac muscle contains approximately more than twofold of *β*1-adrenoceptors than *β*2 [1]. This forms a theoretic basis for adjusting doses of *β*_2_ agonists to affect skeletal rather than myocardial muscles. Indeed, proper dosages of *β*2 agonists, such as clenbuterol, separate the effects on different types of muscles in patients [9–11]. Among the known *β*_2_ agonists, clenbuterol appears to have the safest cardiac profile. To elaborate, clenbuterol given at 1000 *μ*g/kg/day, caused no myocardial hypertrophy in rats, but rather skeletal muscle hypertrophy [32]. Even though extremely high dose of clenbuterol (2000 *μ*g/kg/day) in animals can induce cardiac hypertrophy, that is still physiological in its functions, structure, and gene expressions, it is ultimately free of pathological changes [8, 17, 33]. Consistently, clenbuterol (up to 720 *μ*g/day) promotes cardiac recovery in patients with left ventricular unloading atrophy [12–14]. 

Physiological dose of clenbuterol in rats, 10 *μ*g/kg/day, attenuated denervated muscle atrophy without affecting the heart or causing myocyte death [25, 34]. That dosage was calculated based on the metabolic body weight that 10 *μ*g/kg/day in rats is equivalent to 1 *μ*g/kg/day in humans, a dose safely used in asthma treatments [25, 35]. The dose in the current study was 120 *μ*g/day (~2 *μ*g/kg/day for a 60 kg person). It was well tolerated and not associated with any obvious discomfort, except for one patient with transient nervousness. The newly occurring sinus bradycardia after the clenbuterol trial seemed not be relevant to the activation of the *β*1/2 agonist, which usually leads to tachycardia. Moreover, clenbuterol at the present dose did not exacerbate preexisting minor EKG abnormalities. This is consistent with previous reports that the effects of clenbuterol on the heart are observable at a dose of up to 2100 *μ*g/day in combination therapy for patients using left ventricular assist device. Even at those doses, no severe adverse effects were encountered but tremors and muscle cramps [12, 13]. In our study, no adverse effects on liver, kidneys, lungs or hematopoietic system were observed after the 3-month intake of clenbuterol. 

This pilot single-center study was small in scale. The efficacy would be more apparent in a larger patient population with either gradual increments of clenbuterol doses, or an on and off schedule to avoid the occurrence of receptor desensitization. Recent animal studies also show that clenbuterol is neuroprotective and promotes axon regeneration [5, 28, 29]. Thus, in combination with its antiatrophy function, clenbuterol and its kind truly represent promising and safe agents for countering nerve injuries.

## Figures and Tables

**Table 1:** General characteristics of patients who completed trial.

	EMG			Biopsy		
	Clenbuterol	Placebo		Clenbuterol	Placebo	
Number	15 M = 13, F = 2	17 M = 14, F = 3	*P* value	12 M = 11, F = 1	13 M = 12, F = 1	*P* value

Age, y	27.4 ± 1.6	24.5 ± 2.5	>0.05	28.3 ± 3.1	27.3 ± 1.6	>0.05
Denervation period before trial, m	4.8 ± 0.7	6.5 ± 1.6	>0.05	4.8 ± 1.0	4.7 ± 0.9	>0.05

Figures are means ± standard errors; y: years, m: months.

**Table 2:** Changes of fibrillation potential amplitudes and muscle fibers following treatments.

	Clenbuterol	Placebo	
Decrease in cross-sectional areas of type I fibers, *μ*m^2^	413 ± 91.7	687.1 ± 84.6	*P* = 0.038
Decrease in cross-sectional areas of type II fibers, *μ*m^2^	512.1 ± 75.1	821.3 ± 118.7	*P* = 0.045
Reduction in fibrillation potential amplitude, *μ*V	57.3 ± 32.7	231.5 ± 56.6	*P* = 0.013

Figures are means ± standard errors.
